# The Electroluminescence Mechanism of Solution-Processed TADF Emitter 4CzIPN Doped OLEDs Investigated by Transient Measurements

**DOI:** 10.3390/molecules21101365

**Published:** 2016-10-14

**Authors:** Peng Wang, Suling Zhao, Zheng Xu, Bo Qiao, Zhijuan Long, Qingyu Huang

**Affiliations:** 1 Institute of Optoelectronic Technology, Beijing Jiaotong University, Beijing 100044, China; 15118435@bjtu.edu.cn (P.W.); zhengxu@bjtu.edu.cn (Z.X.); boqiao@bjtu.edu.cn (B.Q.); 13121561@bjtu.edu.cn (Z.L.); 12118413@bjtu.edu.cn (Q.H.); 2 Key Laboratory of Luminescence and Optical Information, Ministry of Education, Beijing Jiaotong University, Beijing 100044, China

**Keywords:** carrier transport and recombination, transient EL, energy transfer, TADF-based OLEDS

## Abstract

High efficiency, solution-processed, organic light emitting devices (OLEDs), using a thermally-activated delayed fluorescent (TADF) emitter, 1,2,3,5-tetrakis(carbazol-9-yl)-4,6-dicyanobenzene (4CzIPN), are fabricated, and the transient electroluminescence (EL) decay of the device with a structure of [ITO/PEDOT: PSS/4CzIPN 5 wt % doped 4,40-N,N0-dicarbazolylbiphenyl(CBP)/bis-4,6-(3,5-di-4-pyridylphenyl)-2-methylpyrimidine (B4PyMPM)/lithium fluoride (LiF)/Al], is systematically studied. The results shed light on the dominant operating mechanism in TADF-based OLEDs. Electroluminescence in the host–guest system is mainly produced from the 4CzIPN emitter, rather than the exciplex host materials.

## 1. Introduction

Organic light emitting devices (OLEDs) have been proven successful in the commercial world, including uses in displays and lighting. If the field continues to progress at its current and rapid pace, OLEDs will soon become a mainstay of our technology. However, the future holds even greater promise for this technology, with an entirely new generation of low-cost, lightweight, and flexible electronic devices on offer. External quantum efficiency (EQE) is one of the key parameters for the application of OLEDs [1]. In an OLED, following electron and hole recombination, two types of excitons—singlets and triplets—are generated at a ratio of 1:3, determined by quantum spin-statistics. Fluorescent OLEDs employ spin-antisymmetric singlets for emission, which provides an internal quantum efficiency limit of 25%, and the remaining 75% of the electrically-generated energy is dissipated via non-radiational methods. As a result, the highest theoretical EQE is restricted to 5% after considering a light out-coupling constant of ∼20% in devices [2,3].

To achieve OLEDs with very high EQEs, many efforts to utilize triplet excitons have been made in order to break the EQE limit of 25%. It has been demonstrated that electro-phosphorescent organic light-emitting devices (PHOLEDs) can yield a 100% internal quantum efficiency [4]. Recently, high-efficiency thermally-activated delayed fluorescent (TADF) emitters have been developed as another way to increase the efficiency of OLEDs [3]. TADF OLEDs harvest both singlet and triplet excitons to provide nearly 100% of the internal conversion of charge into light. In this case, TADF emitters with a small singlet–triplet splitting energy facilitate the thermally-activated reverse intersystem crossing to the singlet manifold, and exerts comparable performance to phosphorescent OLEDs [5,6,7,8]. Kido et al. developed highly-efficient, solution-processed OLEDs using a TADF emitter1,2,3,5-tetrakis(carbazol-9-yl)-4,6-dicyanobenzene (4CzIPN), as a green emitter [9]. The optimized device showed a very low turn-on voltage of 2.5 V at 1 cd/m^2^, and a relatively high EQE of 16%. They observed the formation of exciplex at the host/electron-transporting layer (ETL) interface, which greatly reduced the operating voltage. This work obtained an outstanding efficiency of solution-processed OLEDs. However, the charge transport and recombination processes in this mixed system are still unintelligent, and the performance roll-off was still remarkable under a high current density. Therefore, it is necessary to fully investigate and understand the fundamental mechanisms in this kind of operating OLED in order to achieve a high performance.

Transient electroluminescence (EL) measurement [10] has been proven to be an effective and profitable method for the study of information on injecting carriers. When OLEDs are driven by the pumping signal of the periodic square wave, instead of being driven by DC bias as usual, the devices provide a delayed luminescence after the end of the forward bias pulse, which is known as delayed EL. Transient EL can be used to analyze the formation of excitons in host–guest systems [11], energy transfer processes [12], and carrier transportation [13] in OLEDs. In this work, we have systematically studied a series of highly-efficient, solution-processed TADF OLEDs with the structure ITO/PEDOT: PSS/4CzIPN doped 4,4′-*N*,*N′*-dicarbazolylbiphenyl (CBP, 35 nm)/bis-4,6-(3,5-di-4-pyridylphenyl)-2-methylpyrimidine (B4PyMPM, 55 nm)/lithium fluoride (LiF, 6 Å)/Al (100 nm), and applied transient electroluminescence measurement in order to investigate the fundamental and physical mechanisms of the operating devices.

## 2. Results and Discussion

**Figure 1:**
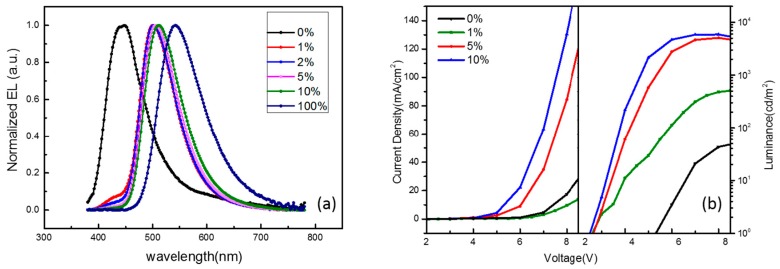
(**a**) The normalized electroluminescence (EL) spectra of devices with different concentrations of 1,2,3,5-tetrakis(carbazol-9-yl)-4,6-dicyanobenzene (4CzIPN) (0% represents 4,4′-*N*,*N′*-dicarbazolylbiphenyl (CBP)-only devices, 100% represents 4CzIPN-only devices, respectively); (**b**) current density–voltage–luminance characteristics of the series of organic light-emitting devices (OLEDs).

**Figure 2:**
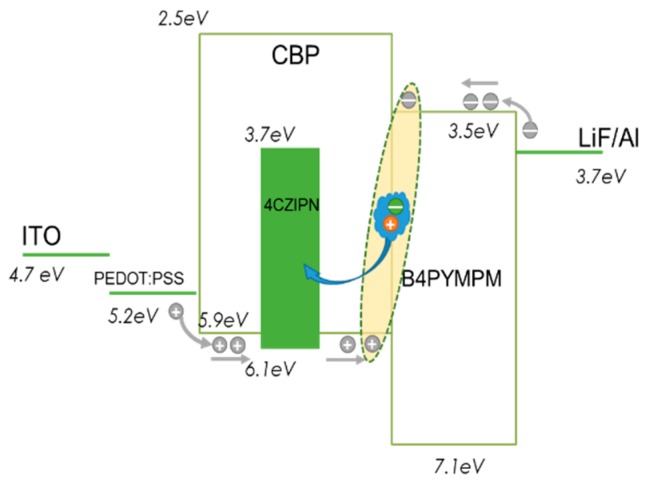
Device structure and energy diagram of organic light emitting devices (OLEDs). B4PyMPM: bis-4,6-(3,5-di-4-pyridylphenyl)-2-methylpyrimidine; ITO: indium tin oxide.

We prepared a guest–host system-based device that avoids concentration quenching, similar to conventional fluorescence and phosphorescence. The host in this system plays a highly important role, because the TADF is an up-conversion of triplet excitons into a singlet state-involved process. The emission spectra of OLEDs containing different concentrations of 4CzIPN are shown in Figure 1a. We can see from the EL spectra that the EL peak of CBP-only devices is about 420 nm, which is identified as the exciplex emission, owing to the absence the EL peak of CBP (about 380 nm) or B4PyMPM (about 460 nm). In the device doped with only 1% 4CzIPN, an emission peak is observed at around 500 nm, and is assigned as the emission from the 4CzIPN molecule. The EL emission of 4CzIPN red-shifts along with the increasing concentration, which is due to the interaction between 4CzIPN molecules. It is noted that a small peak at around 420 nm coinciding with the exciplex emission is observed both in the 1 wt % and 2 wt %-doped devices. This peak decreased with the increasing concentration of 4CzIPN doping, which means an efficient energy transfer from the interface of the CBP/B4PyMPM host to the doped guest. The J-V characteristics of doped and un-doped devices (shown in Figure 1b) are not similar, which suggests that the electroluminescence mechanism is different. As the concentration of 4CzIPN increases, the current density also increases. This means that 4CzIPN acts as a transport carrier. According to the energy level of 4CzIPN (shown in Figure 2), it can transport or trap carriers. If so, the carrier could be recombined directly in 4CzIPN in order to especially emit in devices with a high 4CzIPN concentration, which is different from the results found in Reference [9].

**Figure 3:**
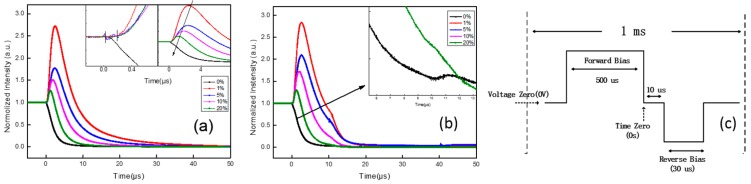
The transient EL curves of 4CzIPN-doped devices with different concentrations; inset is an enlarged view of the transient curves, respectively: (**a**) without a reverse bias pulse; (**b**) with a delay time of 10 μs and a reverse bias of 5 V; (**c**) is the timing scheme used in transient EL measurements.

In order to confirm the electroluminescence mechanism of 4CzIPN, delayed EL measurement was used as an effective method to study the dynamic process of the injecting carriers and excitons in various OLED systems. Measuring the delayed electroluminescence, a persistent EL was found in OLEDs after the electrical bias is turned off, which has been proven to be useful for detecting and identifying various exciton–exciton and carrier–exciton phenomena. In order to enhance the efficient performance and stability of TADF-based OLEDs, it is necessary to gain new insight from the phenomena behind the very high efficiencies attainable in such devices. The time of application of forward and reverse biases is given in Figure 3c; the delayed EL is collected following a 500 μs forward bias square pulse, where the pulse is sufficiently long to allow the prompt EL to reach its steady-state intensity. In Figure 3, a value of 0 on the x-axis corresponds to the time when the forward bias was switched off; this produces the decay luminescence, which is called transient electroluminescence. Generally, the delayed EL in doped OLEDs is raised in the following processes [12,13,14,15]: (1) recombination of trapped charges are released at the end of the forward bias pulse; (2) the slow migration of host excitons to neighboring guest sites, where the energy transfer can take place; and (3) the contribution of triplet-triplet-annihilation (TTA). In our prepared devices, the main electroluminescence mechanism is expected as the carrier recombines directly on the TADF material (4CzIPN) for a high efficiency, but not through the energy transfer of exciplex excitons to 4CzIPN. If so, all carriers injected into 4CzIPN will realize the emission with an efficiency of around 100%. In order to distinguish the definite set of processes occurring in 4CzIPN-based OLEDs, a reverse pulse was applied to prepared devices during the second half of the signal.

Figure 3 shows the transient EL curves of 4CzIPN-doped devices with different concentrations. When driven by a periodic square wave bias (Figure 3a), after the bias is switched off, all devices express obvious spikes, except for the OLED with an emitting layer (EML) made of undoped CBP/B4PyMPM. This means that the electroluminescence of 4CzIPN is not due to the pure energy transfer from the exciplex excitons formed in the interface of CBP/B4PyMPM. Upon closer inspection, with the increase in 4CzIPN concentrations, the peak intensity gradually decreases, and the time needed to reach its peak is shortened. The transient spikein the EL intensity after the bias turn-off is attributed to the release of trapped charges or residual (i.e., accumulated) charges in the EML.

Under low concentrations, exciton recombination on the host material will play a dominant role. Therefore, the EL luminescence of 4CzIPN is mainly from the Förster resonance energy transfer (FRET) of the exciplex excitons formed in the host material. The guest molecules act as carrier traps, and the relative trapped charges are released by the sudden change in voltage and lead to the sudden increase of electron-hole recombination to yield an increase in EL intensity. As the 4CzIPN concentration increases, the exciton formation on the host decreases, the energy transfer from the exciplex is reduced, and the direct recombination of carriers injected into 4CzIPN hold the dominant position. This implies the reduction of carrier traps; therefore, the EL intensity spike is weakened with the increase in 4CzIPN concentration. In addition, the guest molecule concentration increases the probability that the recombination of trapped carriers was increased, leading to the earlier attainment of peak intensity.

As shown in Figure 3b, a reverse pulse with a width of 30 μs was applied to the devices with a delay time of 10 μs behind the forward bias, which is longer than the typical lifetime of singlet excited-state 4CzIPN (~20 ns). The doped devices showed an accelerated EL decay under the reverse bias, compared with Figure 3a, while the undoped device experienced a partial increase when the reverse pulse was on. The joining of the reverse bias sped up the dissociation of the exciton in 4CzIPN formed under a forward bias. If the transient EL is induced by the direct recombination of injected charges, a sudden decrease will appear when the reverse bias is applied; however, there will be no recovery at the ending edge of the reverse pulse compared with TTA [13]. If the exciton of 4CzIPN is formed by the energy transfer from the interfacial exciplex exciton, the transient EL intensity form of the devices doped with 4CzIPN should depend on that of the device without 4CzIPN. The difference in the EL decay from doped and undoped devices means that the energy transfer between the interfacial exciplex to 4CzIPN is not the main process for the emission of 4CzIPN, especially in highly-doped devices. Therefore, it is concluded that the main mechanism of the electroluminescence of the 4CzIPN-doped CBP/B4PyMPM system is the direct recombination of the injected carrier of 4CzIPN.

**Figure 4:**
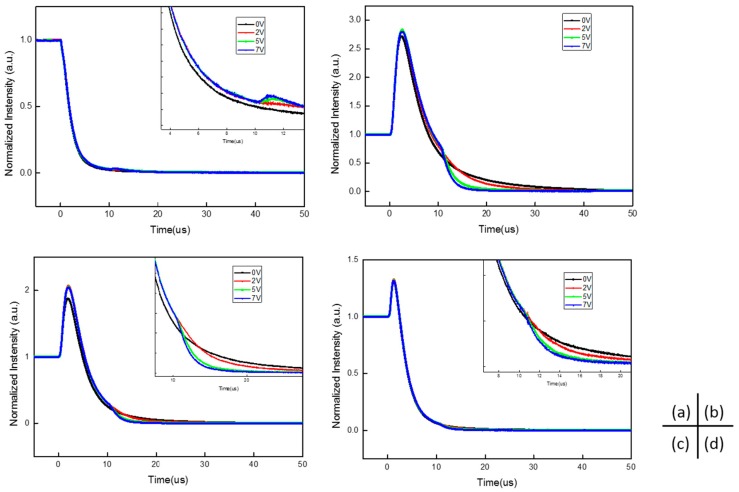
The transient EL curves of 4CzIPN doped devices with different reverse bias; inset is the enlarged view. (**a**) Is for the un-doped devices; (**b**–**d**) are 1%, 5%, and 10% doped devices, respectively.

For further investigation, reverse biases with different magnitudes were applied to these devices, and results are shown in Figure 4. As can be seen in Figure 4a, increasing the magnitude of the reverse bias results in an increase of the spike at the beginning of the reverse bias in the device without 4CzIPN. This suggests that, in exciplex emitting devices, the delayed emission is primarily due to the recombination of released trap charges [12] at the interface between CBP and B4PyMPM, under the applied reverse voltage. In contrast, the increase in reverse bias speeds up the dissociation of excitons from 4CzIPN, causing a decrease in the decay curves in doped devices (shown in Figure 4b–d, respectively. The transient EL of 20 wt % doped devices are not shown here, as the data are similar to those doped at 10 wt %). Based on the systematic investigation of delayed EL, the main mechanism of 4CzIPN emission is due to the carrier recombination of 4CzIPN. The energy transfer process in these doped devices is neglected, especially when the concentration of 4CzIPN is high.

## 3. Experimental Section

The structure of the TADF devices and the principal materials used in this work are shown in Figure 2. CBP, which exhibits bipolar transmission capability, was used as the host for 4CzIPN, a typical TADF molecule [3]. A wide energy gap electron transporter, B4PyMPM, was used as the ETL. The emitting layer (EML) was spin-coated from a chlorobenzene solution (10 mg/mL) in a nitrogen atmosphere; the ETL and cathode were fabricated using thermal evaporation at a deposition rate of 1–2 Å/s in vacuum, with a pressure of about 2 × 10^−4^ Pa. Doping concentrations of 4CzIPN in CBP are 1, 5, 10, and 20 wt %, respectively. The transient electroluminescence properties were detected using a Zolix Instruments Model PMTH-S1C1-CR131 Photomultiplier Tube. The time evolution of delayed light emission was recorded with a Tektronix Model DPO 4104 digital phosphor oscilloscope. All measurements were performed at room temperature under an atmospheric environment.

## 4. Conclusions

We fabricated solution-processed OLEDs using a TADF emitter 4CzIPN-doped CBP/B4PyMPM system. It is worth noting that the formation of exciplex at the host/ETL interface can greatly reduce the operating voltage, and thus significantly improves device performance. We also studied the corresponding transient EL decay of this system. The results provide direct evidence to identify the dominant operating mechanism in TADF-based OLEDs. The main mechanism of the electroluminescence of the 4CzIPN-doped CBP/B4PyMPM system is the injected carrier recombination of 4CzIPN. The energy-transfer process in these doped devices is neglected, especially when the concentration of 4CzIPN is high.
